# Dental publications on the treatment of temporomandibular disorders: A bibliometric analysis

**DOI:** 10.12688/f1000research.177216.3

**Published:** 2026-04-16

**Authors:** Fredy Hugo Cruzado-Oliva, Elmo Linder Cruzado-Oliva, Heber Isac Arbildo-Vega, Franz Tito Coronel-Zubiate

**Affiliations:** 1 Faculty of Stomatology, Stomatology School, Universidad Nacional de Trujillo, Trujillo, La Libertad, 13001, Peru; 2 Faculty of Engineering, School of Industrial Engineering, Universidad Nacional de Trujillo, Trujillo, La Libertad, 13001, Peru; 3 Faculty of Dentistry, Dentistry School, Universidad San Martin de Porres, Chiclayo, Lambayeque, 14012, Peru; 4 Faculty of Health Sciences, Stomatology School, Universidad Nacional Toribio Rodríguez de Mendoza de Amaazonas, Chachapoyas, Amazonas / Chachapoyas, 01001, Peru

**Keywords:** bibliometrics, research, temporomandibular joint, temporomandibular joint disorders, evidence-based dentistry

## Abstract

**Background:**

Temporomandibular disorders (TMD) are a common cause of orofacial pain, and research on therapeutic approaches has grown substantially in recent years. This bibliometric study aimed to assess the scientific output of Scopus-indexed articles on temporomandibular disorder (TMD) treatment, highlighting research trends, key authors, keywords and influential patterns.

**Methods:**

A comprehensive search was conducted in the Scopus database from January 2014 to Au-gust 2024.

**Results:**

The search retrieved 621 articles; after excluding those not related to TMD treatment, 220 were analyzed. The main topics were treatment types and classifications of TMD. The journal contributing the most scientific content was the Journal of Oral and Maxillofacial Surgery. The leading institutions in this field were three universities from Brazil.

**Conclusions:**

The analysis reveals a steady increase in publications related to TMD treatment. These findings describe publication patterns rather than clinical efficacy or quality of evidence. Further systematic evaluations are needed to determine whether these research trends translate into standardized treatment protocols.

## Introduction

Temporomandibular disorders (TMD) are a worldwide public health problem, affecting approximately 5% to 12% of the population. ^1^ It does not distinguish between age and sex, affecting up to 11% in children and 31% in adults, ^2^ and appears to be three times more frequent in women. ^3^ TMD is the second most common musculoskeletal condition after chronic low back pain, with an overall prevalence of 90%, which can affect daily activities, psychosocial functioning and quality of life of the individual. ^1^

TMDs refer to various neuromuscular and musculoskeletal conditions of the temporomandibular joint complex, including surrounding muscular and skeletal structures. ^4^ Its etiology is complex and is widely recognized as multifactorial and biopsychosocial, involving multiple predisposing and precipitating factors related to anatomy, occlusion, parafunction, trauma, and psycho-emotional conditions. ^5^

It is associated with a variety of painful symptoms, such as ear and facial pain, headache in the temporal region and tooth sensitivity, as well as non-painful symptoms such as clicking, popping or crackling of the temporomandibular joint (TMJ), limited jaw movements and muscle fatigue or stiffness. ^6^

The diagnosis of TMD is based on a detailed medical examination, aided by imaging tests. ^4^ Diagnostic criteria with simple, clear, reliable and valid operational definitions are needed for clinical history, examination and imaging procedures to make physical diagnoses in both clinical and research settings. ^7^

In 2014, the Diagnostic Criteria for Temporomandibular Disorders (DC/TMD) were introduced and are now considered the gold standard for the clinical assessment of TMD patients. Based on the patient’s signs and symptoms, the DC/TMD defines two axes. Axis I, categorizing as Group I muscular (including myofascial pain with or without limitation of mouth opening) and arthrogenic TMD; as Group II, disc displacement with or without reduction and limitation of mouth opening and Group III, arthralgia, arthritis and osteoarthritis; Axis II, evaluating disability due to TMD pain through assessment of behavioral and psychological status. This diagnosis is complemented through cone beam computed tomography (CBCT) and magnetic resonance imaging (MRI) examinations. MRI is accepted as the gold standard for evaluating inflammatory conditions and soft tissue areas, including muscles, ligaments, and the cartilaginous disc of the TMJ; on the other hand, CBCT is recommended for evaluating hard skeletal and dental tissues. ^8^

Regarding the treatment of patients with TMD, the main goals to focus on are to reduce TMJ and masticatory muscle pain, improve TMJ function and prevent further TMJ deterioration. ^1^ Current management of TMD follows a hierarchical approach, in which treatment options can be broadly classified into four main categories: (1) conservative non-invasive therapies, (2) pharmacological management, (3) minimally invasive procedures, and (4) invasive surgical interventions. This classification reflects the current clinical recommendation to prioritize reversible and low-risk treatments before considering more invasive approaches.

In this scenario, several therapeutic approaches have been described, including conservative non-invasive therapies, pharmacological management, minimally invasive procedures, and invasive surgical interventions. Non-pharmacological conservative therapies are generally recommended as first-line treatment due to their reversibility and low risk of adverse effects. ^9^

Among conservative non-invasive therapies, several approaches have been described, including educational therapy, cognitive behavioral therapy, manual therapy, occlusal splints, photobiomodulation, and dry needling. These interventions are widely used due to their reversibility and low risk profile and are generally recommended as first-line strategies in TMD management. Previous studies have reported their effectiveness in reducing pain, improving function, and enhancing quality of life in patients with TMD. ^10– 16^

In addition to conservative non-invasive therapies, pharmacological management is frequently used as an adjunct for pain control. Pharmacological treatment can play a crucial role in pain management and improvement of overall quality of life in patients with painful TMD. Fifty percent of patients with painful TMD have reported the use of medications. Nonsteroidal anti-inflammatory drugs (NSAIDs), opioids, corticosteroids, anxiolytics, antidepressants, muscle relaxants and anticonvulsants are the most frequently prescribed pharmacological agents by physicians. ^6^

Minimally invasive procedures include intra-articular and intramuscular injections into the temporomandibular joint (TMJ). The use of botulinum toxin type A (BoNT/A) inhibits presynaptic acetylcholine release at the neuromuscular junction, resulting in a reduction of postsynaptic muscle contraction. It also has an antinociceptive effect, and its main mechanism of action is mediated by the blockade of neuropeptides and the release of inflammatory mediators, in addition to its analgesic effects. ^5, 17^ The injection of hyaluronic acid (HA) is a polymer recognized as a critical component of synovial fluid that lubricates the joints and surrounding tissues. It reduces joint pain by lowering the levels of inflammatory mediators and has long-lasting positive effects. ^18^ Platelet-rich plasma (PRP) injection is a concentrate of platelets suspended in plasma that contains growth factors. These platelets actively secrete protein growth factors that initiate wound healing. It helps restore intra-articular hyaluronic acid and stimulates cartilage cells to produce glycosaminoglycans. It also regulates the balance of angiogenesis within the joint. Moreover, it has anti-inflammatory, antibiotic, and analgesic properties, demonstrating efficacy in the treatment of joint clicking in patients with internal temporomandibular joint disorders. ^19^ Sodium hyaluronate (SH) injection is the main component of synovial fluid and can reduce friction caused by joint movement, lubricate the joints, improve the physiological function of the joints, and protect them through anti-inflammatory mechanisms. SH is the most commonly used drug for intra-articular injections in TMJ treatment. ^20^

When conservative and minimally invasive treatments fail, invasive surgical procedures may be considered. These include minimally invasive arthroscopic procedures or invasive open joint surgeries such as disc plication, discectomy and arthroplasty. ^21^

Bibliometrics is a method for assessing and monitoring the progress of specific disciplines through statistical analysis of published data. It can also be used to determine the results and citations of authors, institutions and countries, and the keyword frequency of hotspots and research frontiers in particular fields. It plays a fundamental role in helping relevant people to categorize research trajectories, discover disciplinary boundaries, and identify research hotspots. ^22^

This research presents a comprehensive bibliometric analysis of current scientific research related to the treatment of TMDs. We explore the evolution of this research area, identifying the main trends, highlighting the best researchers, journals and institutions, and evaluate the influence of this research on the scientific community.

Our goal is to provide a comprehensive overview of how science is addressing this global health problem. By understanding the trajectory and scope of this research, we aim to characterize publication patterns, research trends, and scientific output in this field, providing a structured overview to future bibliometric and evidence-synthesis studies.

## Methods

### Data source and search strategy

**Figure 1.**
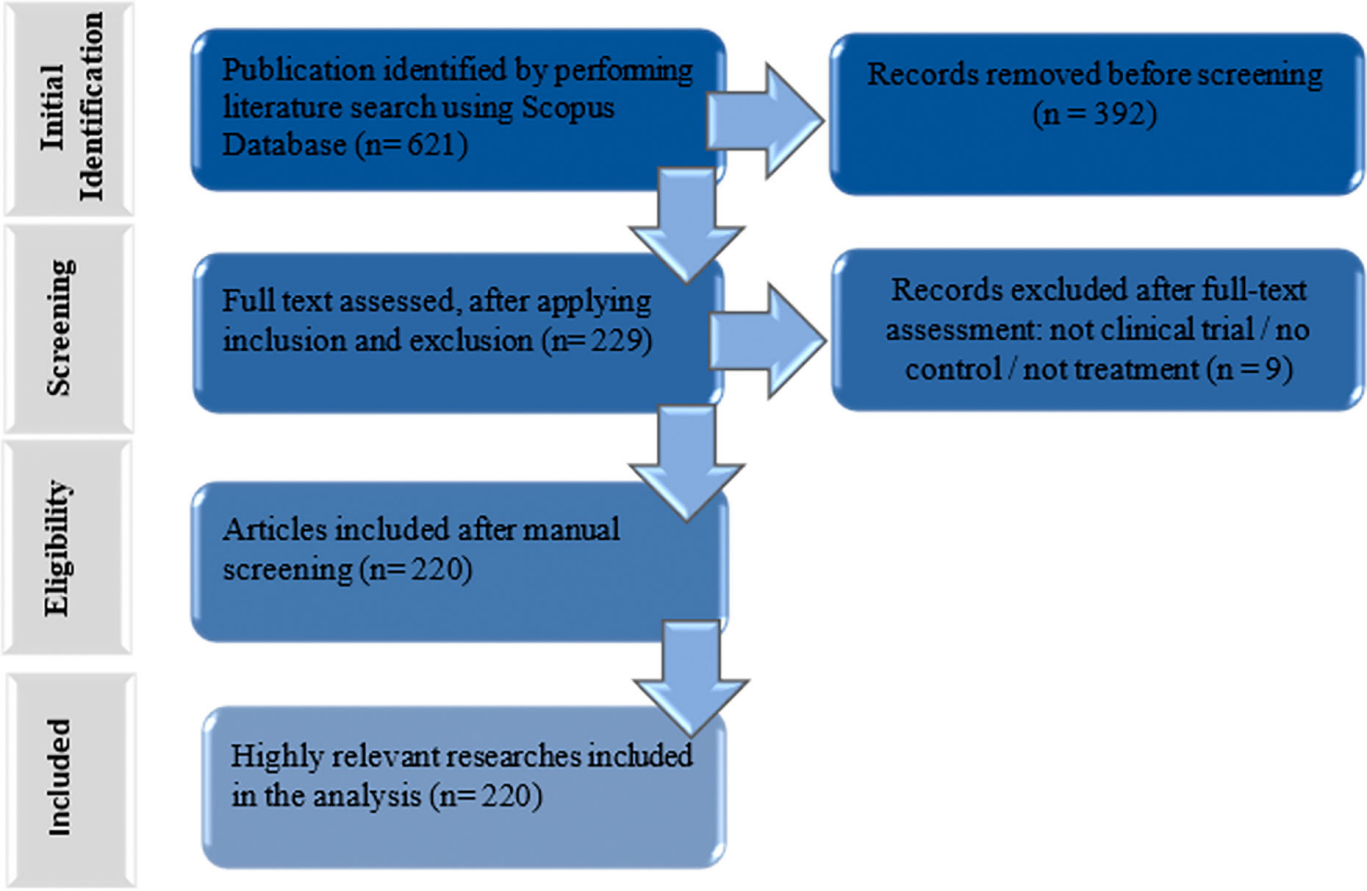
Flow diagram of the study selection process. A total of 621 records were identified through the Scopus database. After removing non-relevant records during the initial filtering stage (n = 392), 229 articles were screened. After full-text assessment, 9 articles were excluded for not meeting the inclusion criteria (e.g., lack of control group or not corresponding to clinical trials), resulting in a final sample of 220 studies included in the bibliometric analysis.

For this analysis, an electronic search was performed for the period from January 2014 to August 2024, using Elsevier’s Scopus database ( https://www.scopus.com), selected for its broad coverage of peer-reviewed journals, comprehensive indexing of citation data, and its suitability for bibliometric analyzes due to standardized metadata and analytical compatibility. The search string used was: (TITLE-ABS-KEY ((“Temporomandibular Joint Dysfunction Syndrome” OR “Temporomandibular Joint Disorders” OR “Temporomandibular Disorder” OR “TMD” OR “Temporomandibular Joint” OR “TMJ”)) AND TITLE-ABS-KEY ((“Clinical Trial” OR “Randomized Clinical Trial”))) AND PUBYEAR > 2013 AND PUBYEAR < 2025 AND (LIMIT-TO (DOCTYPE, “ar”)) AND (LIMIT-TO (EXACTKEYWORD, “humans”)). The selection process was conducted by two independent reviewers. First, duplicate or non-relevant records identified during the initial search were removed automatically by the database filters. Then, titles and abstracts were screened to exclude studies not related to TMD treatment. Full-text assessment was subsequently performed to confirm eligibility according to the inclusion criteria. Any discrepancies between reviewers were resolved through discussion and consensus. In cases where agreement could not be reached, a third author was consulted to ensure consistency in the selection process. Articles were excluded if they did not correspond to clinical trials, did not involve human subjects, or were not directly related to therapeutic interventions for TMD. The complete selection process, including the number of records excluded at each stage, is shown in Figure 1. The full list of selected articles and the selection process are available as extended data (Table S1). ^23^

Inclusion criteria for this bibliometric analysis included a focus on articles without language restriction, but conducted within the last 10 years. In terms of publication type, the search was restricted to clinical trials and randomized clinical trials. To ensure the accuracy of study design classification, the identification of clinical trials was based on both Scopus indexing (document type and keywords) and manual verification during the full-text assessment. Only studies explicitly reporting clinical trial designs involving human participants were included. This decision was made to focus the bibliometric analysis on studies evaluating therapeutic interventions with higher levels of clinical evidence, allowing a more precise assessment of trends in treatment approaches for TMD. Including all types of publications could introduce heterogeneity related to reviews, case reports, or observational studies, which were not the focus of the present analysis. Exclusion criteria included animal studies, literature review, systematic reviews and meta-analyses. Throughout the initial screening process by the investigators, articles were further disqualified if the publications were not related to the treatment domain of TMD. As the research was conducted in October 2024, the analysis was limited to publications indexed between 2014 and 2024, corresponding to a predefined 10-year bibliometric window. This fixed time frame was selected to allow a standardized comparison of publication trends over time. Updating the search to include later publications (2025–2026) would modify the dataset and affect the reproducibility of the bibliometric mapping; Therefore, the predefined time window was maintained for methodological consistency.

### Bibliometric analysis and visualization

Since this research did not involve any human or animal interaction, no ethical approval was required for this analysis. After article selection, data were exported from the Scopus database in BibTeX format. The search results were analyzed using “bibliometrix” ( https://www.bibliometrix.org/home/) which provides the tools to perform a complete bibliometric analysis, following the Scientific Mapping workflow.

VOSviewer software (version 1.6.19) was also used to construct and visualize bibliometric networks. Co-occurrence networks represent how frequently two terms or authors appear together within the same bibliographic records. In the visual maps, nodes represent authors and keywords; lines represent co-occurrence or co-citation; and node sizes were determined by frequency of occurrence while line thickness was determined by the strength of the co-occurrence relationship. The maps present elements that received different colors based on the average year of occurrence, with blue and green elements appearing earlier, and yellow and red elements appearing later.

For the analysis and visualization of the data, we analyzed the evolution of the total publications, the analysis of the treatments performed in each article, the most studied TTMs, the main journals where they were published, as well as the collaboration network between authors, the main academic institutions and the most frequent keywords in the research (2014-2024).

## Results

1. Study selection and general characteristics

This study characterized the scientific output related to TMD treatment research. Numerical data were calculated from the bibliographic characteristics observed in the documents published in the Scopus database. A total of 621 articles were retrieved and 220 were selected based on the selection criteria ( Figure 1). The synthesis of the results and the detailed characteristics of the included studies are provided as extensive data (Table S2). ^23^

2. Publication trends

**Figure 2.**
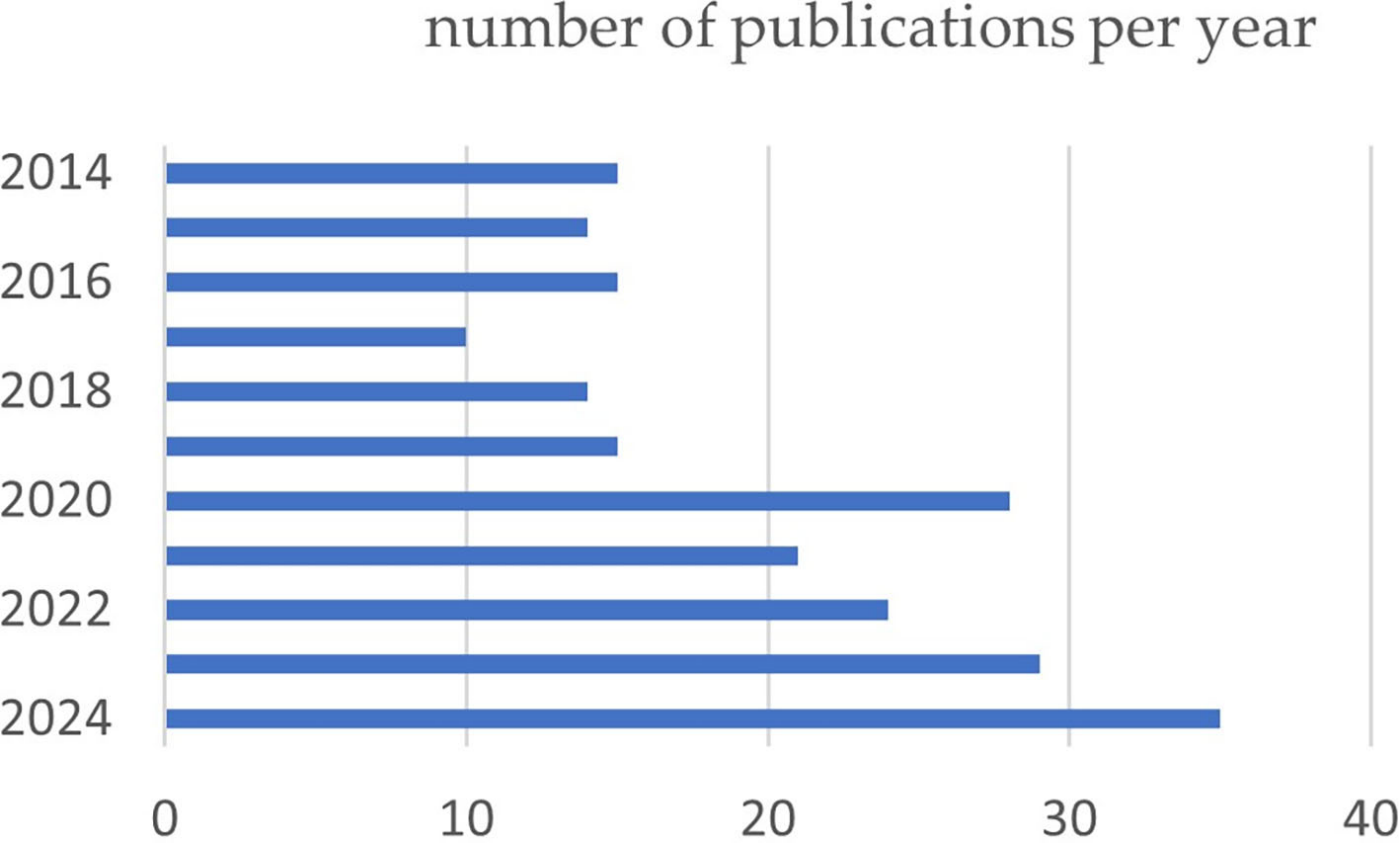
Graph illustrating the trend in the number of articles published over the past 10 years.

During the period analyzed, a variation in the number of articles published can be observed. From 2014 to 2019, the number of publications was moderate, with a maximum of 16 articles in 2016; however, the rate of the number of articles increased progressively from 2020, reaching the maximum number of publications of 35 articles in 2024 ( Figure 2).

3. Citation analysis

**Figure 3.**
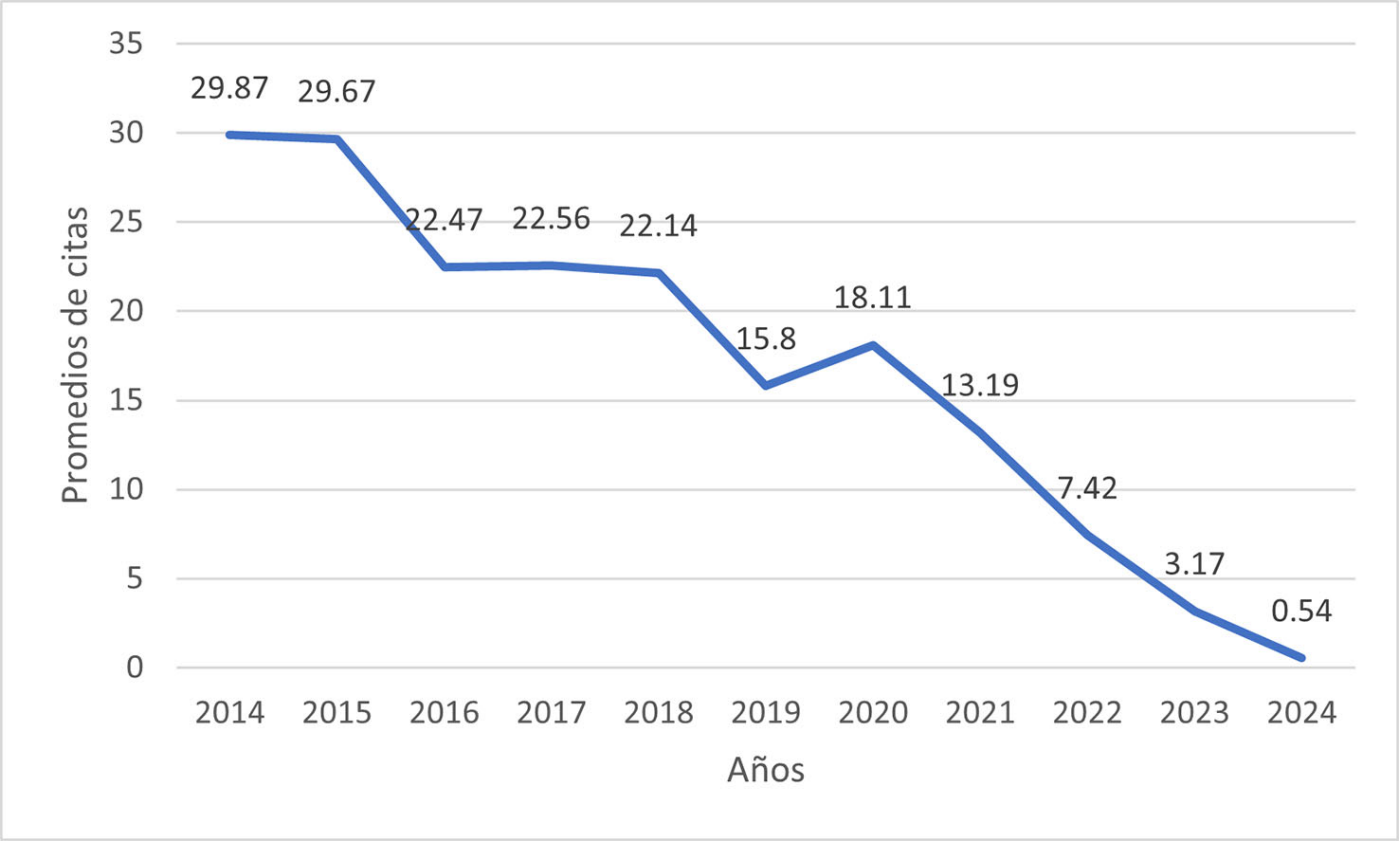
Longitudinal trend of annual citation averages (2014–2024). The graph illustrates a peak in citation impact during the 2014–2015 period (mean > 29 citations/year), followed by a progressive decline in recent years.

Citation-based indicators were calculated to assess the scientific visibility of the retrieved publications. The bibliometric analysis revealed an overall average of 13.86 citations per article during the 2014–2024 period. A clear inverse pattern was observed between publication age and cumulative citation volume. Peak citation rates were recorded in 2014 (29.84 citations/year) and 2015 (29.67 citations/year), followed by a stable plateau between 2016 and 2018 (averaging ~22 citations). Starting from 2019 (15.8 citations), a progressive decline in annual values was identified, reaching minimums in 2023 (3.17) and 2024 (0.54), which is consistent with the time required for the maturation of scientific impact ( Figure 3).

4. Most influential publications

Regarding the influence of individual manuscripts, the work by Ahrari et al. (2014), titled “The efficacy of low-level laser therapy for the treatment of myogenous temporomandibular joint disorder”, was the most cited article with a total of 89 citations (averaging 8.9 per year). This is followed by two clinical trials of substantial relevance published by Cömert et al.: the first (2015) regarding the superiority of arthrocentesis combined with platelet-rich plasma (PRP) (72 citations; 8.0 CPY), and the second (2016) comparing platelet-rich plasma with hyaluronic acid (69 citations; 8.6 CPY).

5. Distribution of treatment modalities

The treatments performed in the different journals analyzed in the last 10 years (2014-2024) were conservative, non-pharmacological treatments with a number of 135 papers, of which 127 had a parallel study design and 123 were randomized; They involved the use of education, self-care, jaw and physical exercises, manual therapies, physiotherapy, acupuncture, transcutaneous electrical nerve stimulation (TENS), occlusal splints (conventional, milled and printed), low-intensity laser (red, infrared and LED), ultrasound and ozone therapy. This was followed by minimally invasive treatments with 66 papers, of which 64 were in parallel and 56 randomized; they consisted of the infiltration of intra-articular substances (hyaluronic acid, platelet-rich plasma, growth factors, stem cells, non-steroidal anti-inflammatory drugs-NSAIDs, corticosteroids, dextrose) and intramuscular (botulinum toxin). The most commonly used conservative pharmacological treatments were NSAIDs and muscle relaxants. Finally, invasive treatments, where all were parallel and randomized, were condylectomy and arthroscopy. For more details on the types of treatment and study characteristics, see Table 1.

**Table 1.** Table detailing the types of treatments and characteristics of the included studies.

Type of treatment		Study design		Randomized studies		Total
		Parallel	Crossover	Yes	No	
Conservative treatment	**Non-pharmacological **	127	8	123	12	135
	**Pharmacological**	13	1	13	1	14
Minimally invasive treatment		64	2	56	10	66
Invasive treatment		5	0	5	0	5
		209	11	197	23	**220**

6. Journals analysis

The number of articles published in different scientific journals between the years 2014 to 2024 regarding TMD treatment research was Journal of oral and maxillofacial surgery with 14 papers, followed by two journals with the same number of papers Lasers in medical science and the Journal of oral rehabilitation with 11. More than 85% of the journals only contributed with 1 paper. For more details on the journals and the number of publications, see Table 2.

**Table 2.** Table listing the journals and the number of studies published in each.

Scientific journals	Articles
Journal of oral and maxillofacial surgery	14
Lasers in medical science	11
Journal of oral rehabilitation	11
Journal of cranio-maxillofacial surgery	10
Cranio - journal of craniomandibular and sleep practice	10
BMC oral health	9
Toxins	7
Cranio - journal of craniomandibular practice	7
Trials	6
Pain research and management	5
Journal of maxillofacial and oral surgery	5
International journal of oral and maxillofacial surgery	5
Medicine (United States)	4
Journal of oral and facial pain and headache	4
British journal of oral and maxillofacial surgery	3
Oral surgery, oral medicine, oral pathology and oral radiology	3
Journal of manipulative and physiological therapeutics	3
Journal of craniofacial surgery	3
Clinical oral investigations	3
Brazilian dental science	3
Acta odontologica scandinavica	2
Bangladesh medical research council bulletin	2
Brazilian oral research	2
Codas	2
Indian journal of dental research	2
Journal of applied oral science	2
Journal of clinical medicine	2
Journal of indian academy of oral medicine and radiology	2
Journal of indian prosthodontic society	2
Journal of photochemistry and photobiology b: biology	2
Oral diseases	2
Pain medicine (united states)	2
Scientific reports	2
Acta clinica croatica	1
Advances in medical sciences	1
Alternative therapies in health and medicine	1
American journal of physical medicine and rehabilitation	1
Annals of anatomy	1
Applied sciences (switzerland)	1
Bioengineering	1
Biomedicines	1
BMC musculoskeletal disorders	1
BMC sports science, medicine and rehabilitation	1
Brain stimulation	1
Brazilian journal of oral sciences	1
Chiropractic and manual therapies	1
Clinical and experimental dental research	1
Comparative exercise physiology	1
Complementary therapies in medicine	1
Dentistry journal	1
Diagnostics	1
Disability and rehabilitation	1
European journal of clinical and experimental medicine	1
European review for medical and pharmacological sciences	1
Evidence-based complementary and alternative medicine	1
Frontiers in dentistry	1
Frontiers in neurology	1
Head and face medicine	1
Healthcare (switzerland)	1
Indian journal of public health research and development	1
International dental journal	1
International journal of environmental research and public health	1
International journal of pharmacy and technology	1
International journal of prosthodontics	1
Jama network open	1
Jams journal of acupuncture and meridian studies	1
Jmir research protocols	1
Journal of back and musculoskeletal rehabilitation	1
Journal of biological regulators and homeostatic agents	1
Journal of biomedical science	1
Journal of bodywork and movement therapies	1
Journal of clinical and diagnostic research	1
Journal of clinical and experimental dentistry	1
Journal of complementary and integrative medicine	1
Journal of headache and pain	1
Journal of indian prosthodontist society	1
Journal of lasers in medical sciences	1
Journal of oral research	1
Journal of orthopaedic and sports physical therapy	1
Journal of photochemistry and photobiology	1
Journal of prosthetic dentistry	1
Journal of research in dental and maxillofacial sciences	1
Journal of stomatology, oral and maxillofacial surgery	1
Journal of taibah university medical sciences	1
Journal of the formosan medical association	1
Journal of ultrasound in medicine	1
Lasers in dental science	1
Life	1
Medical forum monthly	1
Medicina (lithuania)	1
Medicina clinica	1
National journal of maxillofacial surgery	1
Odovtos - international journal of dental sciences	1
Oral and maxillofacial surgery	1
Pain	1
Pediatric rheumatology	1
Photobiomodulation, photomedicine, and laser surgery	1
Photomedicine and laser surgery	1
Physiotherapy theory and practice	1
Pilot and feasibility studies	1
Plastic and reconstructive surgery	1
Scientific world journal	1
Total	**220**

7. Institutional productivity

The institutions that have contributed research on the treatment of temporomandibular disorders are headed by Brazilian universities, University of São Paulo, University of Nove de Julho (UNINOVE) and Federal University of Rio Grande do Sul with 13, 11 and 7 articles respectively, suggesting a strong commitment to research in this area. Followed by institutions from Turkey, University of Gaziantep and Iran, Tehran University of Medical Sciences with 5 articles both. However, there are several other institutions that also have an interest in the subject. For more details on the institutions and the number of publications, see Table 3.

**Table 3.** Table listing the institutions and the number of studies associated with each.

Affiliation	Articles
University of São Paulo, São Paulo, Brazil	13
Nove de Julho University (UNINOVE), São Paulo, Brazil	11
Federal University of Rio Grande do Sul, Porto Alegre, Brazil	7
Gaziantep University, Gaziantep, Türkiye	5
Tehran University of Medical Sciences, Tehran, Iran	5
Ataturk University, Erzurum, Türkiye	4
Istanbul University, Türkiye	4
Federal University of Rio Grande do Norte (UFRN), Natal, Brazil	3
Pomeranian Medical University, Szczecin, Poland	3
Jagiellonian University, Krakow, Poland	3
Mashhad University of Medical Sciences, Iran	3
Cairo University, Cairo, Egypt	3
Xi’an Jiaotong University, China	3
Malmö University, Malmö, Sweden	3
Methodist University of Piracicaba, Brazil	2
Federal University of Santa Maria, Santa Maria, Brazil	2
Federal University of Minas Gerais – UFMG, Brazil	2
Federal University of the Vales of Jequitinhonha and Mucuri, Minas Gerais, Brazil	2
Rio de Janeiro State University, Rio de Janeiro, Brazil	2
University of Campinas, Sao Paulo, Brazil	2
University of Coimbra, Coimbra, Portugal	2
University of Amsterdam, Netherlands	2
University of Milan, Milan, Italy	2
University of Padova, Padova, Italy	2
University of Rome, Rome, Italy	2
Shiraz University of Medical Sciences, Shiraz, Iran	2
Baghdad University, Baghdad, Iraq	2
Silesian Medical University, Katowice, Poland	2
Karadeniz Technical University, Trabzon, Türkiye	2
Afyonkarahisar University of Health Sciences, Afyonkarahisar, Türkiye	2
Turkish University of Health Sciences, Ankara, Türkiye	2
University of North Carolina, North Carolina, USA	2
Emory University, Atlanta, USA	2
King Abdul Aziz University, Saudi Arabia	2
Al-Azhar University, Egypt	2
Tanta University, Tanta, Egypt	2
University of Erlangen Nuremberg, Germany	2
KAHER Institute of Dental Sciences KLE VK, India	2
Tamil Nadu Government Dental College and Hospital, Nadu, India	2
Maulana Azad Institute of Dental Sciences, New Delhi, India	2
SRM Dental Hospital, Chennai, India	2
College of Dental Sciences and Research, Thrissur, Kerala, India	2
Complutense University of Madrid, Madrid, Spain	2
University of Seville, Seville, Spain	2
Andrés Bello University, Viña del Mar, Chile	2
University of Groningen, Netherlands	1
Federal University of Rio de Janeiro, Rio de Janeiro, Brazil	1
Western Paraná State University, Paraná, Brazil	1
Brazilian Lutheran University, Brazil	1
Municipal of Santo Antônio do Pinhal, São Paulo, Brazil	1
Fluminense Federal University, Brazil	1
Pontifical Catholic University of Paraná, Curitiba, Brazil.	1
Federal University of Alfenas, Brazil	1
Federal University of Ceará, Fortaleza, Ceará, Brazil	1
State University of the West of Paraná, Paraná, Brazil	1
University Center of the Hermínio Ometto Foundation, Brazil	1
Federal University of Paraíba, Brazil	1
University of Coimbra, Coimbra, Portugal	1
University of Lisbon, Portugal	1
University of Health Sciences, Gandra, Portugal	1
Egas Moniz Interdisciplinary Research Center, Caparica, Portugal	1
Sassari University Hospital, Sassari, Italy	1
University of Udine, Udine, Italy	1
Vita-Salute San Raffaele University, Milan, Italy	1
“Paolo Giaccone” Polyclinic, Palermo, Italy	1
University of Rome and Eurekacademy ETS, Rome, Italy	1
Shahid Beheshti University of Medical Sciences, Iran	1
Damascus University, Damascus, Syria	1
Gajju Khan Medical College, Swabi, Pakistan	1
Poznan University of Medical Sciences, Poland	1
Ege University, Türkiye	1
Harran University, Sanlıurfa, Türkiye	1
Aichi Gakuin University, Japan	1
National Hospital, Kyoto Medical Center, Japan	1
University School of Dentistry, Nagoya, Japan	1
University of Florida, Gainesville, USA	1
Weill Cornell Graduate School of Medical Sciences, New York, USA	1
Marquette University, Milwaukee, Wisconsin, USA	1
University of Michigan, Michigan, USA	1
University of Washington, Washington, USA	1
Boston University, Boston, USA	1
Baylor University, Waco, Texas	1
University of Zurich, Zurich, Switzerland	1
Taipei Medical University Hospital, Taiwan	1
University of Calabar/Calabar University Hospital, Nigeria	1
Ain Shams University, Egypt	1
Beni-Suef University, Egypt	1
Alexandria University, Alexandria, Egypt	1
Mansoura University, Mansoura, Egypt	1
Heinrich-Heine University Düsseldorf, Germany	1
Maximilian Ludwig University, Munich, Germany	1
Greifswald University, Germany	1
University Hospital, LMU Munich, Munich, Germany	1
Karolinska Institute and Scandinavian Center for Orofacial Neurosciences, Huddinge, Sweden	1
Wenzhou Medical University, Zhejiang, China	1
Sichuan University, Sichuan, China	1
China PLA General Hospital, Beijing, China	1
Chongqing Medical University, China	1
Riphah International University, Lahore, Pakistan	1
Seoul National University, South Korea	1
University of Chile, Santiago, Chile.	1
Daejeon University, Daejeon, Republic of Korea	1
University of Freiburg, Germany	1
University of Delhi, New Delhi, India	1
College of Dental Sciences and SDM Hospital, Karnataka, India	1
Surendera Research Institute, Rajasthan, India	1
Mujib Medical University, Bangladesh, Iraq	1
Government Hospital, Gujarat, India	1
Kalinga Institute of Dental Sciences, Odisha, India	1
Dental Institute, RIMS, Jharkhand, India	1
ATM Consulting Services, Madhya Pradesh, India	1
Government Dental College and Hospital, Mumbai, India	1
Army Hospital, New Delhi, India	1
National Autonomous University of Mexico, Mexico	1
Autonomous University of San Luis Potosí, San Luis Potosí, Mexico	1
Yonsei University, Republic of Korea	1
University of Santiago de Compostela, Spain	1
Abat Oliva University, Barcelona	1
University of Jaén, in Jaén, Andalusia, Spain	1
King Juan Carlos University, Alcorcón, Spain	1
Francisco de Vitoria University, Madrid, Spain	1
University of Salamanca, Salamanca, Spain	1
La Princesa University Hospital, Madrid, Spain	1
Autonomous University of Madrid, Madrid, Spain	1
Stefan cel Mare University of Suceava, Suceava, Romania	1
University of Helsinki, Helsinki, Finland	1
University of Eastern Finland, Finland	1
Lithuanian University of Health Sciences, Kaunas, Lithuania	1
Catholic University of Uruguay, Montevideo, Uruguay	1
Stockholm Public Dental Health, Stockholm, Sweden	1
Umeå University, Umeå, Sweden	1
Zagreb University Hospital Center, Zagreb, Croatia	1
Norwegian Arctic University, Tromso, Norway	1
Total	**220**

8. Co-authorship network analysis

**Figure 4.**
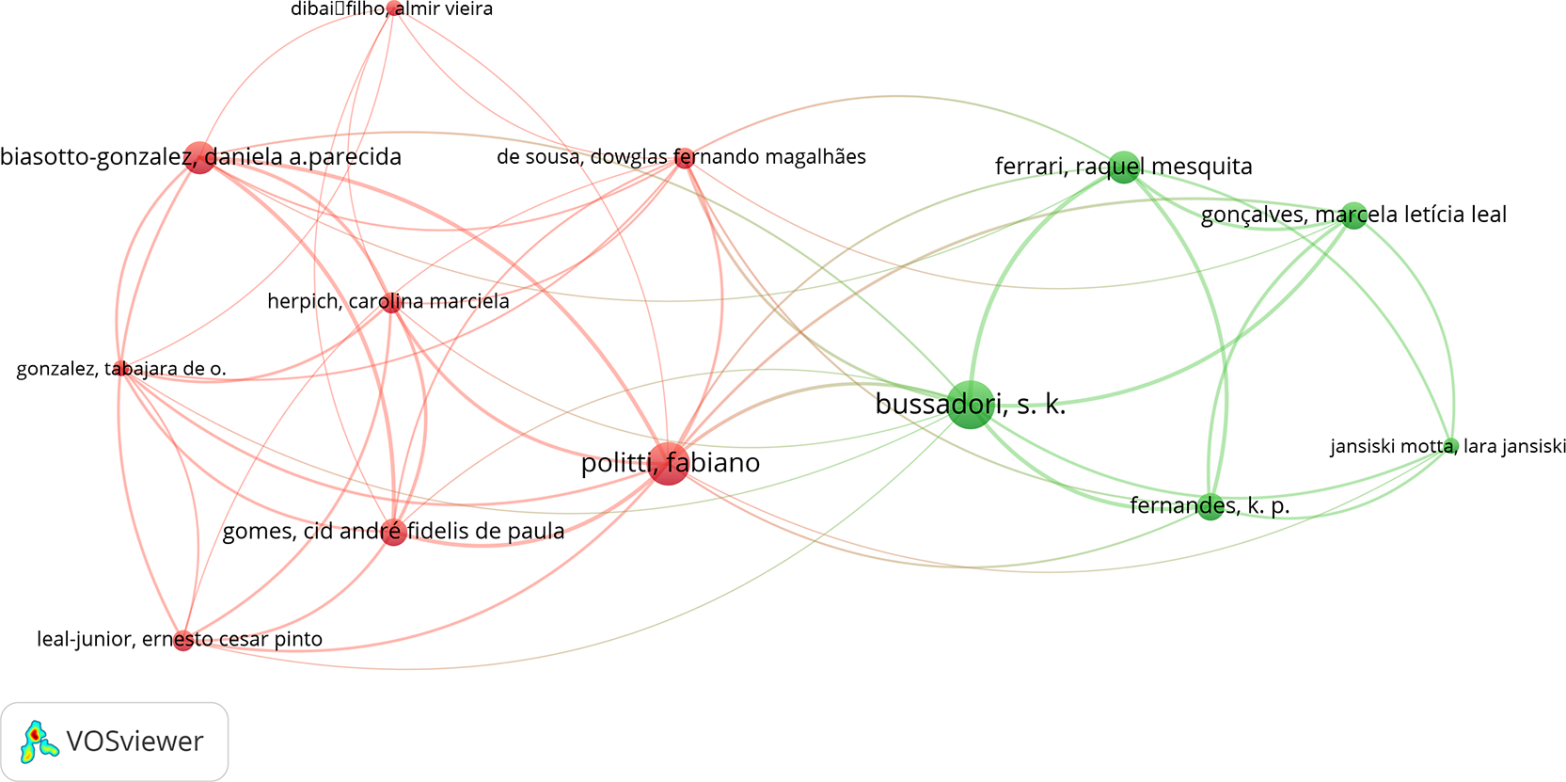
Graph showing co-authorship connections.

The various relationships identified and the nodes in the co-authorship analysis were examined using the VOSviewer software. The analysis showed that the most prominent co-authors were Bussadori, Sandra Kalil; Politti, Fabiano; and Biasotto-Gonzalez, Daniela, with 9, 8, and 6 co-authorships, respectively ( Figure 4).

9. Keyword analysis

**Figure 5.**
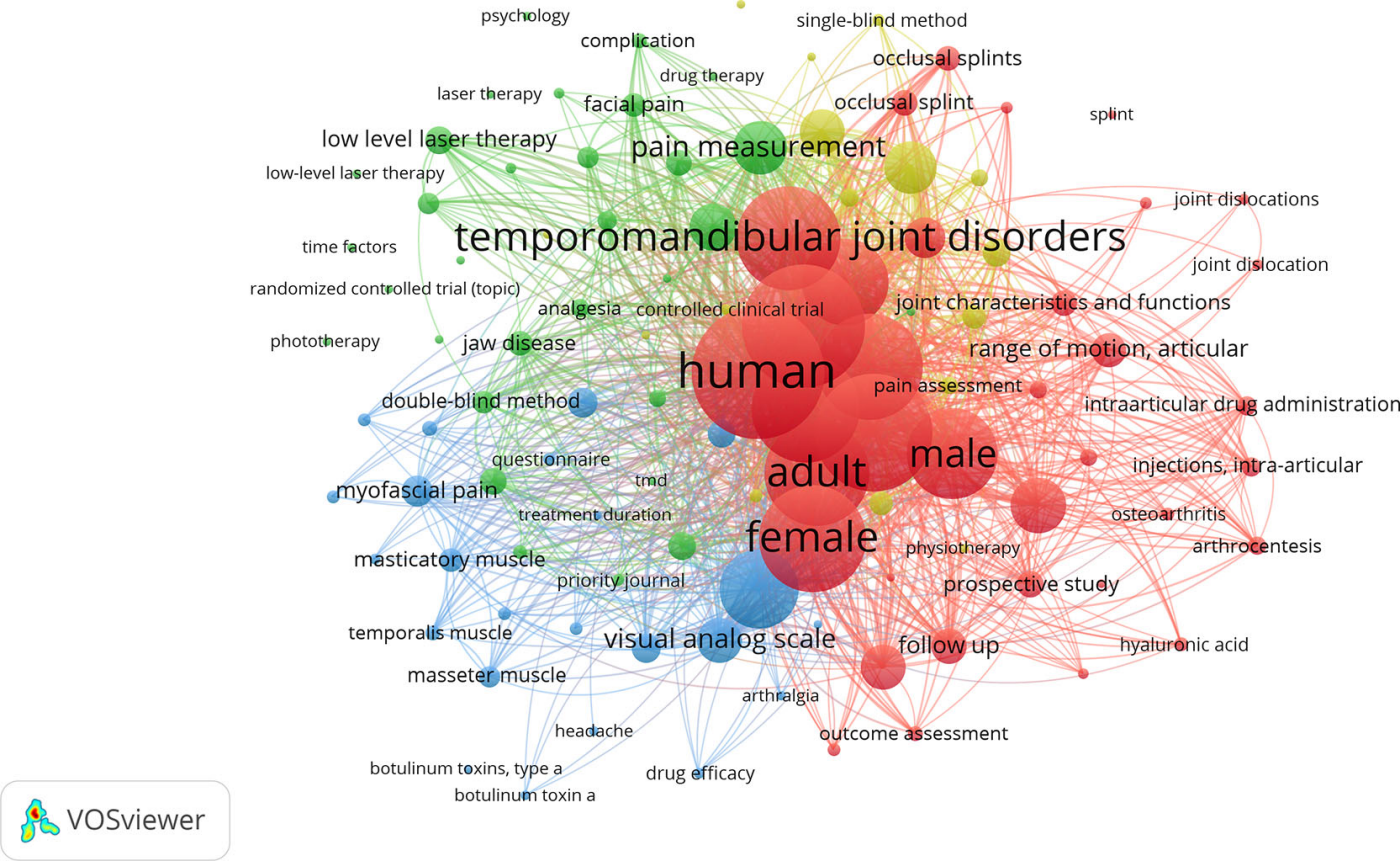
Graph illustrating the most frequently used terms and their connections.

The different relationships and nodes identified in the keyword or term analysis were also examined using VOSviewer. The most frequently used terms were: humans (study population); temporomandibular joint disorder (signs and symptoms related to the TMJ); treatment (outcomes of interventions to reduce TMD); and pain (most common symptom in TMD). The timeline view also reveals the evolution of keywords related to TMD treatment, where blue indicates older terms and red represents more recent or updated ones ( Figure 5).

## Discussion

The present research aimed to evaluate the bibliometric profile of the scientific production, of the documents published between 2014 and 2024 (the last 10 years) on the treatment of TMDs, obtaining as a result 220 documents. The bibliometric indicators were only obtained from the Scopus database, because it provides a broader and more inclusive content coverage; it presents the availability of individual profiles for all authors, institutions and sources of periodicals, as well as the interrelated interface of the database; the implemented impact indicators perform as well or even better than metrics in other databases, are less susceptible to manipulation, and are available for all journal sources in all disciplines; and finally, it is more open to society, as it provides free access to author and source information, including metrics. ^24^

The number of scientific publications in the last 10 years on the treatment of TMDs has increased markedly, with an average of 15 papers in 2014 to 35 papers so far in 2024, but the gradient was not constant throughout all these years. This finding indicates increased publication activity in this area. Similarly, growth in publication volume has also been reported in bibliometric studies in recent years was found in several bibliometric studies relating orthognathic surgery to temporomandibular disorders, ^25^ on the temporomandibular joint and occlusion, ^26^ and on joint disc displacement as the most common TMD condition. ^22^ An increase in publication volume reflects growing academic attention to a topic, although it does not directly indicate clinical effectiveness or evidence quality. ^27^ These publications have changed over the years from conservative or non-invasive therapies such as manual therapy, ^28, 29^ laser therapies, ^30, 31^ occlusal splints, ^32, 33^ acupuncture, ^34, 35^ and use of drugs ^36, 37^; to minimally invasive, such as intra-articular ^38, 39^ or intramuscular ^40, 41^ injection of solutions; and invasive, such as surgical treatments. ^42, 43^

Regarding citation counts, the decrease observed in recent years (2022–2024) likely reflects the typical citation lag inherent to bibliometric cycles. Continued academic interest in the field during this period is reflected by a 133% increase in publication volume compared to 2014, alongside a concentration of research in high-impact, first-quartile (Q1) journals, such as the Journal of Oral and Maxillofacial Surgery and the Journal of Oral Rehabilitation. This trend suggests sustained research activity and increasing scientific interest in this field. Collectively, the three most cited manuscripts represent highly visible contributions within the literature over the last decade, highlighting these therapies as highly represented topics within the most cited literature.

The most frequent treatments found on TMJ in the analyzed papers were conservative non-pharmacological treatments, probably due to their characteristics of being non-invasive, having no known side effects and absence of interactions as in most conservative pharmacological treatments ^15^; furthermore, more than 90% of these clinical trials were in parallel and randomized, suggesting that patients received a single treatment in different groups at random, with the purpose of comparing results. Similar to a bibliometric study conducted in 2024 on the trends and development of articles on the temporomandibular joint. ^44^

Occlusal splint therapy and photobiomodulation were among the most frequently reported interventions. Their prominence in the literature may be related to their accessibility, non-invasive nature, and frequent use in clinical practice. ^45^ However, their high representation should not be interpreted as evidence of superior clinical effectiveness, but rather as an indication of research interest and widespread application.

The high frequency of certain treatments in the analyzed literature should not be interpreted as direct evidence of superior clinical effectiveness. In bibliometric analyses, the number of publications often reflects research interest, accessibility of the technique, and historical use rather than therapeutic superiority. In the case of occlusal splints, their widespread use may be related to their low cost, reversibility, and recommendation as first-line therapy in most clinical guidelines. Similarly, photobiomodulation has gained popularity due to its non-invasive nature and ease of application, although the literature still shows variability in protocols and outcomes.

In addition, many of the reported therapies are not used as isolated interventions in clinical practice, but rather as part of multimodal management strategies. Current approaches to TMD treatment frequently combine patient education, behavioral therapy, pharmacological support, and physical or minimally invasive therapies, following the biopsychosocial model of care. Therefore, the bibliometric predominance of certain treatments should be interpreted within the context of combined therapeutic protocols rather than as independent solutions.

Furthermore, some treatment modalities remain controversial in the literature, with conflicting findings regarding their long-term effectiveness. Differences in study design, patient selection, diagnostic criteria, and outcome measures may explain the variability in reported results. These factors highlight the importance of interpreting bibliometric trends with caution and relating them to current evidence-based clinical recommendations.

The majority of TMD treatments are aimed at relieving the patient’s pain, with more than 50% being muscle type, myalgia (myofascial); followed by the combination of muscle disorders (myalgia) and TMJ disorder (joint pain and/or disc disorder). Pain is the most significant feature of TMD and the main cause for which patients seek treatment, ^46^ the most common type is related to pain in the masticatory, temporal and masseter muscles, with irritable trigger points that become painful with compression; it is aggravated with function, decreasing mouth opening and limiting mandibular movements. ^47^ Thus, these problems directly and negatively influence the physical and mental health of patients, affecting their school, professional and social activities, even causing affective and cognitive imbalance. Therefore, there are detrimental consequences for the quality of life of these people (QOL) and, in particular, for their oral health quality of life (OHQOL), with greater impairments depending on the magnitude of TMD pain. ^48^

This trend suggests sustained research activity and increasing scientific interest in this field. Contemporary clinical guidelines emphasize a conservative and multimodal approach as the first-line strategy, combining patient education, behavioral modification, physical therapy, pharmacological support, and the use of occlusal splints when indicated. The predominance of conservative and minimally invasive therapies in the analyzed literature reflects this clinical preference for reversible and low-risk interventions before considering surgical options.

In addition, the growing interest in non-invasive and combined therapeutic approaches supports the current biopsychosocial model of TMD, in which treatment is directed not only at structural alterations but also at functional, psychological, and behavioral factors. Therefore, the bibliometric patterns identified in this study seem to align with contemporary evidence-based recommendations, although the frequency of publications should not be interpreted as a direct indicator of clinical effectiveness.

Journal quartiles refer to their ranking position within a subject category, while the H-index reflects both publication productivity and citation impact. The most influential journals in scientific production on the treatment of TMDs are the Journal of Oral and Maxillofacial Surgery, with 14 papers, ^41, 49– 61^ is a US journal with an impact factor of 2.1 in 2023, H-index of 140 and is in the first quartile. It is also the official scientific journal of the American Association of Oral and Maxillofacial Surgeons (AAOMS), the American Academy of Craniomaxillofacial Surgery (AACMFS) and the Canadian Association of Oral and Maxillofacial Surgeons (CAOMS); is focused on devel-oping the methods and techniques used in the management of dentoalveolar surgery, facial trauma, deformities, oral cancer, mandibular and facial reconstruction, anesthesia, analgesia and temporomandibular joint (TMJ) disorders.

Most of the analyzed papers used minimally invasive treatments; however, three articles used conservative non-pharmacological treatments; mandibular exercises, ^60^ rigid splint ^54^ and low intensity laser ^51^; and one with invasive treatment, condylectomy. ^59^ The Journal of oral rehabilitation with 11 papers, is a British journal with an impact factor of 3.5 in 2023, an H index of 109 and is also in the first quartile; this journal covers all aspects of oral rehabilitation and applied oral physiology, diagnostic and clinical management aspects necessary to restore harmonious oral function, both subjectively and objectively. In which 8 papers used non-pharmacological conservative treatment; manual therapy, ^62– 64^ splints, ^65– 67^ ozone ^68^ and acupuncture ^69^; one pharmacological conservative treatment ^70^ and two minimally invasive treatment ^71, 72^; probably the large number of papers is due to the mixed scope presented by these journals. However, the journal Lasers in Medical Science with 11 papers, ^73– 83^ is an Iranian journal with an impact factor of 2.1 in 2023, an H-index of 32 and is in the second quartile; of which in all papers they used low level laser therapy, because it is a leading journal in the rapidly expanding field of medical and dental applications, in exclusivity of laser and light. This reflects the thematic focus of these journals on specific treatment modalities.

Out of a total of 133 institutions published relevant literature, it was found that universities produce more scientific publications than institutes, hospitals or private clinics. This is attributed to the physical resources, financial support and variety of cases available at universities compared to hospitals and private clinics. The leading institutions with the most published papers on TMD treatments are the University of São Paulo, Universidade Nove de Julho (UNINOVE) and Federal University of Rio Grande do Sul with 13, 11 and 7 papers respectively; all entities in Brazil. In two bibliometric studies of the Web of Science database, one on articular disc displacement ^22^ and the other on trends and development of ATM articles, ^44^ where they placed the University of São Paulo in third place after the great Asian and American powers, Jiao Tong University of Shanghai, China and University of Rochester, USA. This could be related to the progressive and slow progress of research in Latin America; however, at present it shows us that Brazilian universities have a solid research capacity in this field; in addition to financial resources, university support, international collaborations and the number of academic staff.

The co-occurrence of authorship and its association with other authors were evaluated with the VOS-viewer software, where the author Bussadori, Sandra presents 9 co-authorships, followed by Politti, Fabiano, with 8 and Biasotto-Gonzalez, Daniela, with 6. Where, Bussadori, Sandra shares 3 co-authorships with Politti, Fabiano, one with Bi-asotto-Gonzalez, Daniela and another with both. Politti, Fabiano, shares 4 co-authorships with Biasotto-Gonzalez, Daniela. This suggests the commitment of certain research groups on a certain topic. In addition, we can observe the evolution in time of the re-searches where Politti, Fabiano and Biasotto-Gonzalez, Daniela are in red color which indicates the early start in this field, while Bussadori, Sandra is in green color, which suggests that her participation has been very active in this field in the last years.

The keywords or descriptors were also evaluated by the same software, where the words with the highest scores were temporomandibular joint disorder and human, followed by a medium score temporomandibular joint and treatment or results, and with lower scores pain, adults, masticatory muscles, therapies, etc. This suggests that it is an essential two-way tool for those who write and those who search for information, i.e., it helps to deepen the search for a particular subject area.

Among the main limitations; the data were only collected from Scopus, with the risk of omitting studies included in other databases; due to the limitations of the VOSviewer software, self-citations could not be excluded, which could lead to bias. Another limitation of this study is that the search strategy was restricted to clinical trials and randomized clinical trials. This restriction was applied to focus the analysis on studies with higher levels of clinical evidence related to therapeutic interventions. However, this decision may have excluded other types of publications that could also contribute to understanding the broader research landscape of TMD treatments. Therefore, the results should be interpreted as reflecting trends in clinical intervention studies rather than all available literature on TMD. Finally, the search was limited to the period 2014–2024 because the analysis was designed using a predefined 10-year bibliometric window established at the time of data collection (October 2024). Although updating the search to include more recent publications (2025–2026) could provide additional information, modifying the time frame would affect the consistency of the dataset and the comparability of bibliometric indicators. Therefore, the selected period was maintained to preserve methodological uniformity. However, due to the rapid growth of research on TMD treatments, some recently published studies may not have been included. Periodic updates of bibliometric analyzes are recommended to ensure that future evaluations reflect the most current trends in the field.

## Conclusion

This bibliometric analysis demonstrates a sustained increase in scientific production and a diversification of research topics related to temporomandibular disorder (TMD) treatments over the last decade. The findings provide a structured overview of publication patterns, including the predominance of conservative and minimally invasive approaches, the contribution of leading institutions, and the evolution of research themes.

However, it is important to emphasize that bibliometric indicators reflect research activity and visibility rather than clinical effectiveness or strength of evidence. Therefore, the observed trends should be interpreted with caution and within the context of publication dynamics.

Future research should integrate bibliometric findings with systematic reviews and high-quality evidence syntheses in order to determine whether these trends translate into clinically meaningful improvements in TMD management.

## Ethical considerations

This bibliometric study did not require ethical approval, as it analyzed only data from publicly available literature and did not involve human or animal subjects.

## Data Availability

The bibliometric datasets supporting the findings of this study, including Tables S1 and S2, are publicly available in Zenodo at: https://doi.org/10.5281/zenodo.18250981. ^84^ Zenodo: Bibliometric datasets supporting the findings of this study, including Tables S1 and S2. https://doi.org/10.5281/zenodo.18250981. The dataset is shared under a CC-BY 4.0 license.
