# Supplementary material for: Dental publications on the treatment of temporomandibular disorders: A bibliometric analysis
Source: F1000Res. 2026 Apr 16;15:296. Originally published 2026 Feb 18. [Version 3] doi: 10.12688/f1000research.177216.3 (PMC13047366; doi:10.12688/f1000research.177216.3)
Supplement: Peer review history

# Reviewer report: Reviewer response for version 3

**DOI:** 10.5256/f1000research.198222.r475737

**Published:** 2026-04-24

**Authors:** Brender Leonan-Silva

**Affiliations:** 1 Universidade Federal dos Vales do Jequitinhonha e Mucuri (UFVJM), Diamantina, Brazil

The authors have made substantial improvements to the manuscript and have adequately addressed the main concerns raised in the previous review.

 The Introduction is now clearer and better structured, with appropriate recognition of the multifactorial and biopsychosocial nature of temporomandibular disorders. The Methods section has been significantly strengthened, particularly with the clarification of the study selection process, justification for the use of the Scopus database, and the approach used to identify clinical trials. These changes enhance the methodological transparency and reproducibility of the study.

 The Results section is better organized, and the Discussion shows a more appropriate focus on bibliometric interpretation, including a clearer distinction between research activity and clinical effectiveness. The authors also provided a more critical perspective on the findings, which improves the scientific rigor of the manuscript.

 However, minor revisions are still recommended. Some sections of the Introduction and Discussion remain slightly descriptive and could be further refined to maintain a stronger focus on the bibliometric scope of the study. Additionally, certain parts of the Results could benefit from greater conciseness to improve readability.

 Overall, the manuscript is relevant, methodologically sound, and provides a valuable contribution to the understanding of research trends in temporomandibular disorder treatments.

Is the review written in accessible language?

Yes

Are all factual statements correct and adequately supported by citations?

Partly

Are the conclusions drawn appropriate in the context of the current research literature?

Partly

Is the topic of the review discussed comprehensively in the context of the current literature?

Partly

Reviewer Expertise:

TMDs, Bruxism, Epidemiology

I confirm that I have read this submission and believe that I have an appropriate level of expertise to confirm that it is of an acceptable scientific standard.

# Reviewer report: Reviewer response for version 2

**DOI:** 10.5256/f1000research.197547.r470457

**Published:** 2026-04-02

**Authors:** Ricca Chairunnisa

**Affiliations:** 1 Universitas Sumatera Utar, Medan, Indonesia

- **Is the topic discussed comprehensively?**
*Partly* – the background is strong, but further integration with current literature and clinical perspectives would enhance completeness.
- **Are all factual statements correct and supported?**
*Partly* – most statements are appropriate, though some could benefit from stronger or more recent supporting evidence.
- **Are the conclusions appropriate?**
*Partly* – conclusions are generally supported, but would benefit from clearer emphasis on the descriptive nature of bibliometric findings.

Is the review written in accessible language?

Yes

Are all factual statements correct and adequately supported by citations?

Partly

Are the conclusions drawn appropriate in the context of the current research literature?

Partly

Is the topic of the review discussed comprehensively in the context of the current literature?

Partly

Reviewer Expertise:

Temporomandibular Disorders

I confirm that I have read this submission and believe that I have an appropriate level of expertise to confirm that it is of an acceptable scientific standard.

# Reviewer report: Reviewer response for version 2

**DOI:** 10.5256/f1000research.197547.r469894

**Published:** 2026-03-26

**Authors:** Brender Leonan-Silva

**Affiliations:** 1 Universidade Federal dos Vales do Jequitinhonha e Mucuri (UFVJM), Diamantina, Brazil

Main Comments

 Introduction

 The Introduction remains overly descriptive, with excessive detail on individual therapies. It should be more concise and focused on justifying the bibliometric analysis.

 Additionally, TMD etiology should be described as multifactorial and biopsychosocial (without “probably”).

 Methods

 Methodological details are insufficient. The authors should clarify:

 study selection process (number of reviewers, agreement process),

 justification for using only Scopus,

 and how clinical trials were accurately identified.

 Results

 The Results are mainly descriptive and sometimes difficult to follow. A clearer structure and better organization are needed.

 Discussion

 The Discussion still includes excessive clinical description instead of focusing on bibliometric interpretation.

 More critical analysis is needed, especially regarding research trends and their clinical relevance. Some statements are speculative and should be revised.

 Conclusion

 The study is relevant, but revisions are needed to improve clarity, methodological rigor, and interpretation.

Is the review written in accessible language?

Yes

Are all factual statements correct and adequately supported by citations?

Partly

Are the conclusions drawn appropriate in the context of the current research literature?

Partly

Is the topic of the review discussed comprehensively in the context of the current literature?

Partly

Reviewer Expertise:

TMDs, Bruxism, Epidemiology

I confirm that I have read this submission and believe that I have an appropriate level of expertise to confirm that it is of an acceptable scientific standard, however I have significant reservations, as outlined above.

# Response

**Published:** 2026-03-29

**Authors:** Franz Tito Coronel-Zubiate

**Affiliations:** Public Health, Universidad Nacional Toribio Rodriguez de Mendoza de Amazonas, Chachapoyas, Amazonas, Peru

We would like to thank the reviewer for their constructive and insightful comments. We have carefully revised the manuscript in accordance with the suggestions provided, with the aim of improving clarity, methodological transparency, and the overall scientific quality of the study. Below, we provide a point-by-point response to each comment.

**Comment 1:**

*Introduction*

*The Introduction remains overly descriptive, with excessive detail on individual therapies. It should be more concise and focused on justifying the bibliometric analysis.*

*Additionally, TMD etiology should be described as multifactorial and biopsychosocial (without “probably”).*

**Response:**

 We thank the reviewer for this important observation. The Introduction has been further revised to improve conciseness and focus. Specifically, detailed descriptions of individual therapies have been consolidated into a single paragraph to avoid fragmentation and excessive clinical detail. The section now emphasizes a structured overview of TMD management strategies, aligned with the purpose of the bibliometric analysis.

 In addition, the wording related to TMD etiology has been corrected to reflect the current scientific consensus, removing the term “probably” and clearly stating that TMD is a multifactorial and biopsychosocial condition.

**Comment 2:**

*Methods*

*Methodological details are insufficient. The authors should clarify: study selection process (number of reviewers, agreement process), justification for using only Scopus, and how clinical trials were accurately identified.*

**Response:**

 We thank the reviewer for this important observation.

 The Methods section has been further revised to improve methodological transparency. Specifically, we have clarified that the study selection process was conducted by two independent reviewers, with discrepancies resolved through discussion and consensus, and involvement of a third author when necessary.

 In addition, we have expanded the description of the search strategy to justify the exclusive use of the Scopus database, highlighting its suitability for bibliometric analyses.

 Finally, we have clarified how clinical trials were accurately identified, indicating that study design classification was based on both Scopus indexing and manual verification during full-text assessment.

 These additions strengthen the reproducibility and methodological rigor of the study.

**Comment 3:**

*Results*

*The Results are mainly descriptive and sometimes difficult to follow. A clearer structure and better organization are needed.*

**Response:**

 Response:

 We thank the reviewer for this important comment.

 The Results section has been revised to improve clarity, structure, and readability. Specifically, the section has been reorganized using thematic subheadings to provide a clearer presentation of the findings, including study selection, publication trends, citation analysis, treatment distribution, journal analysis, institutional productivity, and network analyses.

 In addition, descriptive results have been separated from interpretative statements. Any speculative or explanatory content has been removed or relocated to the Discussion section to maintain consistency with reporting standards for bibliometric analyses.

 These modifications improve the organization and facilitate the interpretation of the results.

**Comment 4:**

*The Discussion still includes excessive clinical description instead of focusing on bibliometric interpretation.*

*More critical analysis is needed, especially regarding research trends and their clinical relevance. Some statements are speculative and should be revised.*

**Response:**

 We thank the reviewer for this valuable comment.

 The Discussion section has been revised to improve its focus on bibliometric interpretation and reduce excessive clinical description. Specifically, detailed clinical explanations that were not directly related to the interpretation of bibliometric findings were removed or condensed.

 In addition, the discussion was strengthened by incorporating a more critical analysis of research trends, emphasizing that publication frequency reflects research interest and accessibility rather than clinical effectiveness. Statements that could be considered speculative were revised to ensure a more cautious and evidence-based interpretation.

 Furthermore, greater emphasis was placed on linking bibliometric findings with current clinical approaches, particularly the role of multimodal and conservative management strategies in TMD, while clearly acknowledging the limitations of bibliometric data in reflecting clinical outcomes.

 These changes improve the clarity, scientific rigor, and interpretive depth of the manuscript.

**Comment 5:**

*Conclusion*

*The study is relevant, but revisions are needed to improve clarity, methodological rigor, and interpretation.*

**Response:**

 We thank the reviewer for this comment.

 The Conclusion has been revised to improve clarity, methodological precision, and interpretive balance. Specifically, we have clarified the contribution of the study as a bibliometric analysis, emphasizing that the findings reflect publication patterns rather than clinical effectiveness or quality of evidence.

 In addition, the conclusion now highlights the relevance of integrating bibliometric data with future systematic reviews and evidence-based analyzes to better understand the clinical implications of these research trends.

 These revisions strengthen the scientific rigor and coherence of the manuscript.

# Reviewer report: Reviewer response for version 1

**DOI:** 10.5256/f1000research.195404.r462665

**Published:** 2026-03-14

**Authors:** Brender Leonan-Silva

**Affiliations:** 1 Universidade Federal dos Vales do Jequitinhonha e Mucuri (UFVJM), Diamantina, Brazil

Dear Authors,

 Thank you for the opportunity to review this manuscript, which presents a bibliometric analysis of clinical trials addressing treatments for temporomandibular disorders (TMD). The topic is relevant, and the manuscript provides a useful overview of research activity in this field over the past decade. However, several methodological and structural issues should be addressed to improve clarity, transparency, and the overall scientific contribution of the study.

 Below are specific comments and suggestions for improvement.

 1. Search period and update of the literature

 The study aims to analyze publications from the last ten years, and the search was limited to the period 2014–2024. While the search date (October 2024) explains this timeframe, the manuscript is now being considered in a later publication context. Considering the rapidly expanding literature on TMD treatments, particularly in recent years, an update of the search to include 2025 and possibly early 2026 publications would strengthen the relevance and timeliness of the study.

 If updating the search is not feasible, the authors should clearly justify this limitation in the methods and discussion sections.

 2. Structure and focus of the Introduction

 The Introduction currently presents several treatment modalities in a fragmented manner, with multiple paragraphs describing individual interventions (e.g., education, cognitive behavioral therapy, laser therapy, pharmacological approaches, injections, etc.).

 This structure weakens the narrative flow and partially overlaps with what should be explored in the Discussion.

 I suggest restructuring the Introduction to:

- provide a general overview of TMD management strategies,

briefly mention the main categories of treatment, such as:

1. conservative non-invasive therapies,
2. pharmacological management,
3. minimally invasive procedures,
4. surgical interventions.
5. Specific therapies could then be explored more thoroughly in the Discussion, particularly when interpreting the bibliometric findings.

3. Terminology regarding TMD etiology

 The Introduction states that the etiology of TMD is “probably multifactorial and biopsychosocial.”

 However, current scientific consensus generally recognizes TMD as a multifactorial and biopsychosocial condition, rather than a hypothetical assumption. Revising this wording would improve conceptual accuracy.

 4. Transparency of the study selection process

 Although this study is a bibliometric analysis rather than a systematic review, the flow diagram describing the selection process lacks sufficient transparency.

 The transition between the initially identified records and the final sample of included articles is not clearly explained. In particular, it would be helpful to indicate:

1. how many articles were excluded after title/abstract screening,
2. how many were excluded after full-text assessment,
3. the reasons for exclusion at each stage.

Providing these details would improve methodological transparency and reproducibility.

 5. Search strategy and study design restrictions

 The search strategy appears to restrict the results to clinical trials or randomized clinical trials.

 This methodological decision should be clarified and justified. Bibliometric studies often aim to capture broader patterns in the literature, and limiting the search exclusively to clinical trials may exclude relevant research on TMD treatments.

 The authors should explain the rationale for this restriction and discuss its potential impact on the interpretation of the results.

 6. Interpretation of treatment-related findings

 The manuscript identifies the most frequently studied treatment modalities in the analyzed literature. However, the discussion could be strengthened by exploring the clinical implications of these findings in greater depth.

 For example, when discussing treatments such as occlusal splints or photobiomodulation, it would be valuable to consider:

1. whether their high frequency reflects greater clinical effectiveness,
2. whether they are typically used as isolated therapies or as part of multimodal management,
3. whether there are controversies or conflicting findings in the literature.

This type of analysis would help connect the bibliometric results with clinical relevance and evidence-based practice.

 7. Integration between bibliometric findings and clinical context

 Finally, the discussion could benefit from a more explicit connection between the bibliometric trends identified and the current clinical understanding of TMD management.

 For instance, contemporary guidelines often emphasize multimodal and conservative approaches, including patient education, behavioral strategies, physiotherapy, and splint therapy. Reflecting on whether the bibliometric patterns align with these clinical principles would add interpretative depth to the study.

 The manuscript addresses an important topic and provides a valuable overview of research trends in TMD treatment. However, improvements in methodological transparency, conceptual clarity, and discussion depth are necessary to strengthen the contribution of the study.

 I encourage the authors to address the points raised above to enhance the clarity and impact of the manuscript.

Is the review written in accessible language?

Yes

Are all factual statements correct and adequately supported by citations?

Partly

Are the conclusions drawn appropriate in the context of the current research literature?

Partly

Is the topic of the review discussed comprehensively in the context of the current literature?

Partly

Reviewer Expertise:

TMDs, Bruxism, Epidemiology

I confirm that I have read this submission and believe that I have an appropriate level of expertise to confirm that it is of an acceptable scientific standard, however I have significant reservations, as outlined above.

# Response

**Published:** 2026-03-18

**Authors:** Franz Tito Coronel-Zubiate

**Affiliations:** Public Health, Universidad Nacional Toribio Rodriguez de Mendoza de Amazonas, Chachapoyas, Amazonas, Peru

We sincerely thank the reviewer for the careful reading of our manuscript and for the constructive and valuable comments. We have revised the manuscript accordingly and believe that these suggestions have significantly improved the clarity and quality of the paper.

 Below, we provide a detailed, point-by-point response to each comment. All modifications have been incorporated into the revised manuscript.

**Comment 1:**

*1. Search period and update of the literature*

*The study aims to analyze publications from the last ten years, and the search was limited to the period 2014–2024. While the search date (October 2024) explains this timeframe, the manuscript is now being considered in a later publication context. Considering the rapidly expanding literature on TMD treatments, particularly in recent years, an update of the search to include 2025 and possibly early 2026 publications would strengthen the relevance and timeliness of the study.*

*If updating the search is not feasible, the authors should clearly justify this limitation in the methods and discussion sections.*

**Response:**

 We thank the reviewer for this important observation.

 The search period (2014–2024) was predefined because the bibliometric analysis was designed using a standardized 10-year time window, and the data collection was completed in October 2024. Updating the search to include 2025–2026 publications would modify the dataset and affect the reproducibility and comparability of the bibliometric mapping.

 To clarify this point, we have added an explicit justification in the Methods section and expanded the limitation statement in the Discussion, acknowledging that recently published studies may not have been included and that periodic updates are recommended.

**Comment 2:**

*2. Structure and focus of the Introduction*

*The Introduction currently presents several treatment modalities in a fragmented manner, with multiple paragraphs describing individual interventions (e.g., education, cognitive behavioral therapy, laser therapy, pharmacological approaches, injections, etc.).*

*This structure weakens the narrative flow and partially overlaps with what should be explored in the Discussion.*

*I suggest restructuring the Introduction to:*

- *provide a general overview of TMD management strategies,*

*briefly mention the main categories of treatment, such as:*

1. *conservative non-invasive therapies,*
2. *pharmacological management,*
3. *minimally invasive procedures,*
4. *surgical interventions.*
5. *Specific therapies could then be explored more thoroughly in the Discussion, particularly when interpreting the bibliometric findings.*

**Response:**

 We thank the reviewer for this valuable suggestion.

 Following the recommendation, the Introduction has been reorganized to provide a clearer overview of TMD management strategies.

 Treatment approaches are now presented using a hierarchical structure, grouping them into four main categories: conservative non-invasive therapies, pharmacological management, minimally invasive procedures, and invasive surgical interventions.

 The individual therapies are still briefly described to provide clinical context, but the narrative flow has been improved to avoid fragmentation and to better distinguish the background section from the Discussion.

**Comment 3:**

*3. Terminology regarding TMD etiology*

*The Introduction states that the etiology of TMD is “probably multifactorial and biopsychosocial.”*

*However, current scientific consensus generally recognizes TMD as a multifactorial and biopsychosocial condition, rather than a hypothetical assumption. Revising this wording would improve conceptual accuracy.*

**Response:**

 We thank the reviewer for this important clarification.

 The wording has been revised to reflect the current scientific consensus that TMD has a multifactorial and biopsychosocial etiology, rather than presenting it as a hypothetical assumption.

 The corresponding sentence in the Introduction has been modified accordingly.

**Comment 4:**

*4. Transparency of the study selection process*

*Although this study is a bibliometric analysis rather than a systematic review, the flow diagram describing the selection process lacks sufficient transparency.*

*The transition between the initially identified records and the final sample of included articles is not clearly explained. In particular, it would be helpful to indicate:*

1. *how many articles were excluded after title/abstract screening,*
2. *how many were excluded after full-text assessment,*
3. *the reasons for exclusion at each stage.*

*Providing these details would improve methodological transparency and reproducibility.*

**Response:**

 We thank the reviewer for this helpful comment.

 The flow diagram has been revised to improve transparency in the selection process.

 The updated figure now shows the number of records removed before screening, the number assessed for eligibility, the number excluded after full-text evaluation, and the final number of included studies, together with the reasons for exclusion.

 In addition, the Methods section has been clarified to describe the selection process in detail.

**Comment 5:**

*5. Search strategy and study design restrictions*

*The search strategy appears to restrict the results to clinical trials or randomized clinical trials.*

*This methodological decision should be clarified and justified. Bibliometric studies often aim to capture broader patterns in the literature, and limiting the search exclusively to clinical trials may exclude relevant research on TMD treatments.*

*The authors should explain the rationale for this restriction and discuss its potential impact on the interpretation of the results.*

**Response:**

 We thank the reviewer for this important observation.

 The search strategy was intentionally restricted to clinical trials and randomized clinical trials in order to focus the bibliometric analysis on studies evaluating therapeutic interventions with higher levels of clinical evidence.

 This approach allowed a more precise assessment of trends in treatment modalities for TMD.

 We agree that this restriction may exclude other types of publications, and this has now been clarified in both the Methods and Discussion sections as a methodological limitation.

**Comment 6:**

*6. Interpretation of treatment-related findings*

*The manuscript identifies the most frequently studied treatment modalities in the analyzed literature. However, the discussion could be strengthened by exploring the clinical implications of these findings in greater depth.*

*For example, when discussing treatments such as occlusal splints or photobiomodulation, it would be valuable to consider:*

1. *whether their high frequency reflects greater clinical effectiveness,*
2. *whether they are typically used as isolated therapies or as part of multimodal management,*
3. *whether there are controversies or conflicting findings in the literature.*

*This type of analysis would help connect the bibliometric results with clinical relevance and evidence-based practice.*

**Response:**

 We thank the reviewer for this valuable suggestion.

 The Discussion has been expanded to better interpret the clinical implications of the bibliometric findings.

 We have clarified that the frequency of certain treatments in the literature does not necessarily reflect superior clinical effectiveness, and that many therapies are commonly used as part of multimodal management strategies rather than as isolated interventions.

 In addition, we have discussed the presence of controversies and variability in the literature, emphasizing the need to interpret bibliometric results in the context of evidence-based clinical practice.

**Comment 7:**

*7. Integration between bibliometric findings and clinical context*

*Finally, the discussion could benefit from a more explicit connection between the bibliometric trends identified and the current clinical understanding of TMD management.*

*For instance, contemporary guidelines often emphasize multimodal and conservative approaches, including patient education, behavioral strategies, physiotherapy, and splint therapy. Reflecting on whether the bibliometric patterns align with these clinical principles would add interpretative depth to the study.*

**Response:**

 We thank the reviewer for this insightful comment.

 The Discussion has been expanded to better relate the bibliometric findings to the current clinical understanding of TMD management.

 A new paragraph has been added to explain that the predominance of conservative and minimally invasive treatments in the literature is consistent with contemporary clinical guidelines, which recommend a multimodal and reversible approach as first-line management.

# Reviewer report: Reviewer response for version 1

**DOI:** 10.5256/f1000research.195404.r462669

**Published:** 2026-03-04

**Authors:** Ramesh Patra

**Affiliations:** 1 Lovely Professional University, Punjab, India

This manuscript presents a bibliometric analysis of clinical trial publications on the treatment of temporomandibular disorders (TMD) indexed in Scopus between 2014 and 2024. The authors analyzed 220 eligible articles and examined publication trends, treatment types, journals, institutional productivity, co-authorship networks, and keyword occurrence using Bibliometrix and VOSviewer. The study reports an increase in publications over time, highlights the predominance of conservative non-pharmacological treatments, and identifies Brazilian institutions as leading contributors.

 The topic is relevant and timely, and the use of bibliometric tools is appropriate. The inclusion of visual network analyses and public data availability are strengths. However, several issues require revision.

 Regarding accessibility, the manuscript is partly written in accessible language. While suitable for a specialist dental or bibliometric audience, it contains technical terminology (e.g., co-occurrence networks, H-index, quartile ranking) that may not be clear to broader readers. In addition, there are grammatical inconsistencies and terminology errors (e.g., “Crusader” instead of “Crossover”) that reduce clarity. The authors should define technical terms at first mention and undertake professional language editing.

 Regarding the conclusions, they are partly appropriate. The descriptive findings (e.g., increase in publication volume, dominance of conservative treatments) are supported by the data. However, some conclusions extend beyond what bibliometric analysis can support. Increased publication numbers do not necessarily indicate scientific progress or improved treatment protocols. The manuscript should avoid causal interpretations and limit conclusions strictly to publication trends rather than clinical effectiveness or quality of evidence.

 To make the article scientifically sound, the following points must be addressed:

1. Revise the conclusions to avoid causal or clinical overreach and ensure they remain within the scope of bibliometric analysis.
2. Correct factual inaccuracies and terminology errors.
3. Improve language clarity and consistency through thorough editing.
4. Expand the limitations section to discuss database restriction (Scopus only), search strategy constraints, and potential indexing bias.
5. Strengthen the bibliometric analysis by including citation-based indicators (e.g., total citations, most cited articles, average citations per year) to support claims of influence and impact.

In summary, the manuscript has a solid foundation and addresses an important topic, but it requires substantial revision in interpretation, analytical depth, and language clarity before it can be considered scientifically robust.

Is the review written in accessible language?

Partly

Are all factual statements correct and adequately supported by citations?

Partly

Are the conclusions drawn appropriate in the context of the current research literature?

Partly

Is the topic of the review discussed comprehensively in the context of the current literature?

Partly

Reviewer Expertise:

Bibliometric research, oral health research, temporomandibular disorders (TMD), clinical trial methodology, evidence-based physical therapy, craniosacral therapy, and cranio-cervical osteopathic techniques.

I confirm that I have read this submission and believe that I have an appropriate level of expertise to confirm that it is of an acceptable scientific standard, however I have significant reservations, as outlined above.

# Response

**Published:** 2026-03-08

**Authors:** Franz Tito Coronel-Zubiate

**Affiliations:** Public Health, Universidad Nacional Toribio Rodriguez de Mendoza de Amazonas, Chachapoyas, Amazonas, Peru

We sincerely thank Reviewer 1 for the careful and constructive evaluation of our manuscript entitled “Dental publications on the treatment of temporomandibular disorders: A bibliometric analysis.” We greatly appreciate the positive assessment of the relevance of the topic, the appropriateness of the bibliometric tools, and the strengths of the visual network analyzes and data availability.

 We have carefully revised the manuscript in accordance with all the comments provided. In particular, we improved language clarity and consistency throughout the text, corrected terminology and factual inaccuracies, revised statements that could be interpreted as causal or clinically overreaching, expanded the limitations section, and strengthened the bibliometric analysis by incorporating citation-based indicators, including average citations per article, annual citation trends, and the most cited articles in the dataset. We also added brief explanations of technical bibliometric terms where appropriate to improve accessibility for a broader readership.

 Below, we provide a point-by-point response to the reviewer's main observations.

**Comment 1:**

*Regarding accessibility, the manuscript is partly written in accessible language. While suitable for a specialist dental or bibliometric audience, it contains technical terminology (e.g., co-occurrence networks, H-index, quartile ranking) that may not be clear to broader readers. In addition, there are grammatical inconsistencies and terminology errors (e.g., “Crusader” instead of “Crossover”) that reduce clarity. The authors should define technical terms at first mention and undertake professional language editing.*

**Response:**

 Thank you for this important observation. We have thoroughly revised the manuscript to improve language clarity, consistency, and readability. Terminology errors were corrected, including the replacement of “Crusader” with “Crossover” in Table 1. In addition, technical bibliometric terms such as co-occurrence networks, H-index, and quartile ranking are now briefly explained at first mention to make the manuscript more accessible to non-specialist readers. The text was also carefully edited to correct grammatical inconsistencies and improve overall flow.

**Comment 2:**

*Regarding the conclusions, they are partly appropriate. The descriptive findings (e.g., increase in publication volume, dominance of conservative treatments) are supported by the data. However, some conclusions extend beyond what bibliometric analysis can support. Increased publication numbers do not necessarily indicate scientific progress or improved treatment protocols. The manuscript should avoid causal interpretations and limit conclusions strictly to publication trends rather than clinical effectiveness or quality of evidence.*

*Revise the conclusions to avoid causal or clinical overreach and ensure they remain within the scope of bibliometric analysis.*

**Response:**

 We agree with the reviewer and thank you for highlighting this important issue. The Abstract, Discussion, and Conclusion sections were revised to avoid causal or clinical overinterpretation. We now explicitly state that the findings describe publication patterns and bibliometric trends, rather than clinical efficacy, therapeutic superiority, or quality of evidence. Statements implying scientific or clinical progress were removed or reformulated to remain within the proper scope of bibliometric analysis.

**Comment 3:**

*Correct factual inaccuracies and terminology errors.*

**Response:**

 Thank you. We reviewed the manuscript carefully and corrected factual and terminology-related issues identified during revision. This included typographical and wording corrections, standardization of terminology, correction of institutional names, and refinement of bibliometric expressions to improve scientific precision.

**Comment 4:**

*Improve language clarity and consistency through thorough editing.*

**Response:**

 Thank you. We performed a comprehensive language review across the manuscript to improve clarity, grammar, sentence structure, and consistency of terminology. We also simplified several sentences and refined transitions between sections to make the text more readable.

**Comment 5:**

*Expand the limitations section to discuss database restriction (Scopus only), search strategy constraints, and potential indexing bias.*

**Response:**

 We appreciate this suggestion and have expanded the Limitations section accordingly. The revised now manuscript explicitly acknowledges the exclusive use of the Scopus database, the possibility that some relevant studies indexed only in other databases may have been missed, the constraints associated with the selected search terms and filters, and the potential indexing bias related to database coverage. We also retained the limitations associated with self-citation exclusion and the time lag in citation accumulation and database indexing.

**Comment 6:**

*Strengthen the bibliometric analysis by including citation-based indicators (e.g., total citations, most cited articles, average citations per year) to support claims of influence and impact.*

**Response:**

 Thank you for this valuable recommendation. We have strengthened the bibliometric analysis by incorporating citation-based indicators into the Results and Discussion sections. Specifically, we added the overall average citations per article, the annual trend of citation averages, and a description of the most cited articles in the dataset. We also included a new figure showing the longitudinal trend of annual citation averages and discussed these findings in the context of citation lag and bibliometric visibility. These additions provide a more robust assessment of scientific visibility and influence within the analyzed literature.

**Final response**

 We are grateful for the reviewer's thoughtful comments, which significantly improved the rigor, clarity, and interpretive precision of the manuscript. We believe that the revised version now better reflects the scope and contribution of a bibliometric study and addresses all concerns raised.
